# PROMPT to improve speech motor abilities in children with cerebral palsy: a wait-list control group trial protocol

**DOI:** 10.1186/s12883-022-02771-6

**Published:** 2022-07-06

**Authors:** S. Fiori, C. Ragoni, I. Podda, A. Chilosi, C. Amador, P. Cipriani, A. Guzzetta, G. Sgandurra

**Affiliations:** 1 IRCCS Stella Maris Foundation, Pisa, Italy; 2 Department of Clinical and Experimental Medicine, University of Pisa, Pisa, Italy; 3 Parole al Centro Studio di Logopedia, Genoa, Italy

**Keywords:** Dysarthria, Cerebral palsy, PROMPT, Motor speech treatment, Kinematic, Children

## Abstract

**Background:**

Children with cerebral palsy (CP) often have communication impairments, including speech altered intelligibility. Multiple levels of disrupted speech have been reported in CP, which negatively impact on participation and quality of life, with increase of care needs. Augmentative Alternative Communication (AAC) is an option, with debated benefits and limitations, in particular for its functional use. This is supported by a substantial lack of defined evidences in favor of direct speech articulation intervention in CP. Motor learning-based interventions are effective in CP and are the basis of speech motor interventions such as PROMPT (Prompts for Restructuring Oral Muscular Phonetic Targets). The PROMPT speech motor treatment provides tactile-kinesthetic inputs to facilitate articulatory movements by dynamic modelling, resulting in more efficient motor patterns that can be integrated into speech and communication. In CP, exploratory evidences support the feasibility and preliminarily advantages on intelligibility of motor speech treatments, such as PROMPT, with increased speech motor control, also documented by kinematic analyses.

**Methods:**

A randomized waitlist-control trial will be conducted in children aged between 3- and 10-years having CP and dysarthria (estimated sample size = 60 children). Children will be allocated in the immediate intervention or in the waitlist control group. The intervention consists of an intensive 3 weeks period of twice-a-day administration of PROMPT. Standard care will be administered in the control (waitlist) group. After repeated baseline assessments (T0), the PROMPT treated group will undergo the experimental 3-week intervention period, with T1 assessment at the end. A further T2 assessment will be provided at medium term (3 months after the end of the intervention) for evaluating the stability of intervention. Primary and secondary speech clinical and kinematics outcome measures will be collected at T0, T1 and T2.

**Discussion:**

This paper describes the study protocol consisting of a RCT with two main objectives: (1) to evaluate the or short-term benefits of an intensive speech motor intervention on speech and intelligibility in children with CP and the stability of the intervention at medium term; (2) to describe the kinematic correlates of speech motor control modifications.

**Trial registration:**

Trial registration date 06/12/2019; ClinicalTrials.gov Identifier: NCT04189159.

## Background

Cerebral palsy (CP) is the most common cause of motor disability worldwide, with a prevalence of 2–2.5 per 1000 live births [1]. Several comorbidities distinguish the clinical picture of children with CP, such as communication impairment, feeding difficulties, intellectual disability, vision and hearing impairment and epilepsy. Communication difficulties in CP include altered phonation, low speech intelligibility, abnormal development of gesture and facial expression and receptive and expressive language impairment. Communication impairment has been identified in at least 40% of children with CP [2], with 36–90% of the children undergoing motor speech impairments [3–8]. Recent findings report up to 78% of dysarthria among children with CP and motor speech impairment [9]. However, multiple levels of disrupted speech, involving the development of sounds (articulation), the rules of sound combinations (phonology), phonatory support and the precise execution of speech movements (dysarthria) and their planning/programming (apraxia of speech) can be co-existing in CP [9], thus supporting the potential theoretical value of different intervention approaches and strategies [10, 11]. It has also been suggested that some children with CP who do not present clear symptoms of dysarthria, may still have speech motor control deficits [9, 12, 13]. Poor intelligibility due to motor speech impairments has a strong negative impact on daily life and personal autonomies, it reduces the quality of life and spreads daily care needs [7, 8]. Impaired speech abilities with severe reduction of intelligibility and effortful production are considered as a clue for augmentative alternative communication (AAC) to supplement or replace oral speech in CP, with debated advantages and limitations over AAC functional use [5]. The use of AAC approaches is in part supported by a substantial lack of evidences in favor of direct speech articulation interventions in children with CP [11], despite some preliminary advantages and a possible impact on intelligibility [11, 14].

Only few studies addressed the topic of providing evidence based, reproducible and targeted early interventions to improve speech motor abilities of children with CP. Though the limited evidences on efficacy compared to no treatment at all, it might be hypothesized that in general, motor learning principle-based interventions [15], are indicated for CP to improve also speech intelligibility, voice loudness and articulatory accuracy [14–16]. For instance, in a group of 7 children with spastic quadriplegia, Boliek and Fox [17] described some positive effects on vocal functioning, with limited stability of the benefits on intelligibility by using a motor speech treatment originally developed for adults with Parkinson disease, the Lee Silverman Voice Treatment (LSVT LOUD) [18, 19]. Interestingly, the behavioral changes described following this type of treatment were supported by changes in white matter integrity, as documented through a neuroradiological study of connectivity [20]. Less research has been dedicated to treatment protocols targeting the articulatory subsystem though there is clear evidence of substantial alterations at this level. Ward and colleagues [21] studied the effect of PROMPT (PROMPTs for Restructuring Oral Muscular Phonetic Targets) on articulation in a small group of children aged 3–11 years with different types of CP (dyskinetic, spastic, unilateral and bilateral). This approach, that is consistent with Dynamic Systems Theory (DST), proved effective in changing the speech motor patterns of children with CP, resulting in improved intelligibility and motor quality as documented by kinematic analyses [21]. At our knowledge, the previous papers are among the limited number of studies that investigated the effects of a treatment approach aimed at modifying articulation rather than the subsystems providing respiratory and phonatory support. The few studies that investigated the speech of children with CP through kinematic analyses [12, 22–24] support the presence of a malfunctioning speech motor control, particularly evident when the children’s speech motor systems are challenged by longer sequences and an increasing motor load. This is in agreement with the hypothesis of altered functional synergies involved in speech output in CP, due to abnormal muscular synergies and reduced sensory feedback to motor commands [12]. These physio-pathological hypotheses may suggest a potential therapeutic value of treatment protocols whose main target is the articulatory subsystem and which use tactile-kinesthetic inputs to enhance the speech motor skills of children with CP. Indeed tactile-kinesthetic support, such as provided in the PROMPT treatment [25–27], could enhance the sensory feedback and help to both constrain and facilitate the articulators’ movements through the dynamic modelling of more efficient motor patterns that can be integrated into language and, ultimately, into communication.

Based on previous literature and theoretical assumptions, we hypothesize that children with CP and dysarthria will benefit from a high intensity motor speech treatment based on PROMPT. For this purpose, we designed a wait-list control study by comparing a three-week intensive PROMPT speech motor treatment to standard care. Outcome measures will include motor speech clinical assessment and intelligibility rating, as well as objective kinematic measures of speech motor movements. The stability of the functional improvements will be assessed at medium term.

## Methods/design

### Study design

We plan a randomized trial on the effects of PROMPT therapy in children with CP using a wait-list control group (Table 1). Enrolled children will be randomized into either immediate treatment or wait-list-control group. This design allows every child meeting inclusion criterion to eventually receive the treatment, thus avoiding issues of equipoise.

**Table 1:** Trial detailed information

Data category	Information
Primary registry and trial identifying number	ClinicalTrials.gov Identifier: NCT04189159
Date of registration in primary registry	06 December, 2019
Source(s) of monetary or material support	The Prompt Institute, USA
Primary sponsor	The Prompt Institute, USA
Contact for public queries	SF, MD, PHD simona.fiori@fsm.unipi.it
Contact for scientific queries	SF, MD, PHD simona.fiori@fsm.unipi.it IRCCS Fondazione Stella Maris, Pisa, Italy
Public title	PROMPT to Improve Speech Motor Abilities in Children with Cerebral Palsy: a wait-list control group trial
Scientific title	PROMPT to Improve Speech Motor Abilities in Children with Cerebral Palsy: a wait-list control group trial
Countries of recruitment	Italy
Health condition(s) or problem(s) studied	Speech motor intervention in children with cerebral palsy
Intervention(s)	Active comparator: *PROMPT speech motor intensive trial*
	Standard care
Key inclusion and exclusion criteria	Ages eligible for study: 3–10 years Sexes eligible for study: both Accepts healthy volunteers: no
	Inclusion criteria: infant and children aged 3–10 years; cerebral palsy; speech motor deficit with dysarthria; intellectually adequate or mildly disabled; adequate or mildly impaired comprehension skills; phonatory control/phonate on demand; Italian first or main spoken language
	Exclusion criteria: exclusive use of alternative augmentative communication; medical fragility of structural/anatomical malformations affecting speech production or preventing participation to intervention
Study type	Interventional
	Allocation: randomized intervention model. Single blind (outcomes assessor)
	Primary purpose: improve speech intelligibility
Date of first enrolment	February 2020
Target sample size	60
Recruitment status	Recruiting

**Fig. 1:**
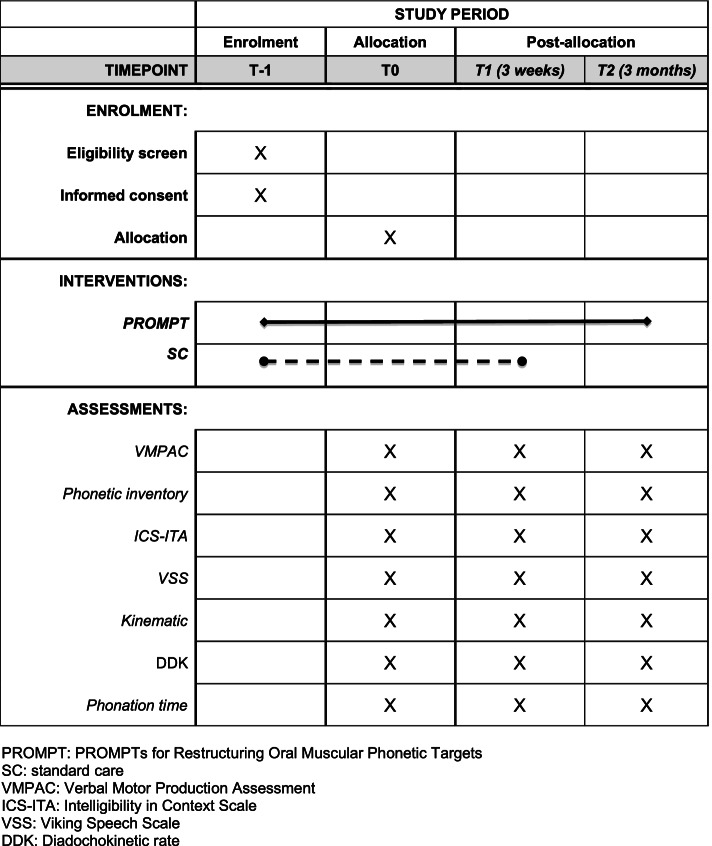
Schedule of study enrolment, interventions, and assessments. PROMPT: PROMPTs for Restructuring Oral Muscular Phonetic Targets. SC: standard care. VMPAC: Verbal Motor Production Assessment. ICS-ITA: Intelligibility in Context Scale. VSS: Viking Speech Scale. DDK: Diadochokinetic rate

Baseline assessment will be carried out after enrollment (T-1) and allocation (T0). In order to obtain reliable baseline speech motor measures, three subsequent assessments of verbal motor skills will be administered at T0 according to the evaluation protocol described in the *Intervention* section. Post-intervention assessment will be administered within 1 week from the end of the treatment (T1). A further stability effect assessment will be performed 3 months from post intervention assessment (T2) in the intervention group for stability assessment. The baseline and outcome assessments will be video recorded for subsequent analyses at each time point (T0, T1 and T2). An experienced speech and language pathologist (SLP), different from the treating therapist will score each time point assessments for data collection. The detail of the assessments per timeline is reported in Fig. 1.

### Participants

Parents will be asked to consent for the children participation to the study. All study activities will be carried out in the clinical and research institute, IRCCS Stella Maris Foundation, Pisa, Italy, by a PROMPT trained therapist (CR) with appropriate knowledge of the clinical research process.

Inclusion criteria: 1) age between 3 and 10 years; 2) a diagnosis of CP; 3) presence of speech motor deficits consistent with dysarthria; 4) normal nonverbal Intelligence Quotient (IQ) or mild intellectual disability with adequate or mildly impaired language comprehension skills 5) presence of volitional phonatory control and the ability to phonate on demand 6) Italian as the first or main spoken language in the child’s environment. Exclusion criteria: 1) use of alternative augmentative communication strategies as the only means of communication, 2) structural anatomical malformations impacting speech production, 3) medical fragility preventing the participation in the intervention.

### Sample size

Based on the yearly number of children referred to the recruiting center for clinical management and rehabilitation, we expect to reach a convenient final sample of 60 subjects with CP and motor speech disorders (including intervention and wait-list) over a 2 years period, by considering a conservative 20% attrition rate for clinical trials. Recruitment will be completed according to the standards of research consent, followed by group randomization by a team member other than the SLPs in charge of treatment and assessment.

### Randomization/blinding

Enrolled participants will be randomly assigned to immediate treatment or waitlist by the use of a random-generator software by a member of the research team specifically designed. The SLP who will score the recorded three timeline for primary outcome measures, as well as the researcher who will analyze kinematic measures will both be blinded to group assignment.

### Study procedure

Eligible children will be referred to the research team for recruitment. The investigators will provide parents/guardians information about the study procedures and request informed consent. Demographic data and other clinical measures, including brain Magnetic Resonance Imaging (MRI) data and co-morbidities, will be collected after participants’ parents/caregivers have provided informed consent. Assessments and procedures included in the study will be provided at IRCCS Stella Maris Foundation, Pisa, Italy.

### Intervention

Each child will receive a full cycle of PROMPT therapy lasting 3 weeks, with two sessions per day over five consecutive days each week. Each session will last 45 minutes. The treatment will be delivered by an experienced SLP with fidelity level adherence to PROMPT [28] treatment criteria and supervised by a PROMPT certified instructor (IP) In PROMPT intervention, goals are chosen to reflect the complex interactions among the physical-sensory, cognitive-linguistic, and social-emotional domains, consistently with the PROMPT systemic conceptual framework. The Motor Speech Hierarchy (MSH) is the speech production and intervention model used to select speech motor goals for intervention. These hierarchical speech motor goals are embedded into the cognitive-linguistic and social-emotional goals for the child. Visual, auditory and tactile-kinesthetic-proprioceptive cueing techniques are used to model speech production and to train the best possible speech motor patterns. In particular, tactile-kinesthetic inputs are used to enhance the sensory input and to facilitate the formation of sensory–motor pathways required for the acquisition and accurate production of speech movement patterns. The principles of motor learning, such as a session of blocked practice followed by randomized practice and considerations on the type of feedback provided to the child (Knowledge of Performance, and/or Knowledge of Results) are applied to intervention sessions depending on the child’s needs. The blocked practice session is generally delivered at the beginning of the session and then is followed by two-three interactive play-based activities during which the selected speech targets are practiced in a randomized fashion and in a functional interactive context. The blocked practice session can be divided into shorter pre-practice sessions delivered two-three times at different points over the treatment session in consideration of the age and compliance of the children. The interactive activities selected for the randomized practice are chosen based on the motivation and interests of the children and are specifically selected and/or adapted to meet their needs in terms of accessibility and ease of manipulation. Adaptive seating is provided for children with more severe challenges in postural control.

### Primary outcome measures

*Verbal Motor Production Assessment for Children (VMPAC)* [29]*:* a standardized motor speech assessment, which includes 5 subscales, where higher scores mean better performance: Global motor control (range 0–20); Focal oromotor control (range 0–26); Sequencing (range 0–46); Connected speech and language (range 0–45); Speech Characteristics (range 0–7). In Global Motor Control the structural and neuromuscular integrity of the oro-facial district, tone and strength are assessed. Focal oromotor control is the area in which motor control in speech and non-speech movements is assessed according to a developmental hierarchical model of speech motor control. Focal oromotor control in the VMPAC is evaluated in movements requiring control of jaw, labial-facial musculature and tongue on only one of the three planes of movements (vertical, horizontal and anterior-posterior) that define the functional space in which speech movements are executed. In the sequencing area, the VMPAC assesses the child’s ability to learn and control non-speech as well as speech sequences across several repetitions of the same targets. In Connected Speech and Language Control the quality of motor control is evaluated during the production of phrases in a picture description task. This area allows for the evaluation of movement patterns that occur during language production and of the interactions between language complexity and the increase of motor load in longer units. Speech Characteristics is an area in which the management of voice parameters, speech rate, prosody and resonance are taken into consideration.

*Phonetic Inventory*: repetition of 21 syllables containing all the Italian consonantal sounds. The children’s performances on this task will be compared to the reference data collected from a group of 40 typically developing (TD) children [30, 31].

*The Intelligibility in Context Scale - Italian version (ICS-ITA)* [32–34]. The ICS provides a speech intelligibility measure with ordinal scores ranging between 1 and 5, where higher scores mean better performances. The scale assesses the intelligibility in functional communication across different settings (home, school, peers, familiar and non-familiar people interactions). Thus, the ICS aligns with the Environmental Factors described in the International Classification of Functioning, Disability and Health: Children and Youth Version (ICF-CY, World Health Organization [WHO]).

*Viking Speech Scale (VSS)* [4, 35], an ordinal scale for intelligibility, with scores ranging between 1 and 4. Lower scores correspond to better performances. The VSS reliably classifies the speech performance of children with CP and is focused on the presence and the severity of the motor speech disorder affecting oral communication with familiar and non-familiar speakers. This scale was developed in the context of a 3 years research program sponsored by the European Community, aiming at the promotion of best practices in the classification of children with CP and at documenting variations in access to health care and in health outcomes.

### Secondary outcome measures

*Kinematic speech motor measures*. A kinematic analysis of facial movements during simple speech repetition tasks will be implemented ad hoc, by means of a non-invasive marker procedure. Such analyses aim to record the variations over time of 5 different metrics quantifying the movements of jaw and lips [12], thus assessing the mandibular and labial-facial control pre- and post-intervention. The procedure will be conducted as follows. The subjects will sit in front of a video screen and will be asked to produce the words included in a probe words list, either by spontaneously naming the pictures presented on the screen or on repetition of the examiner’s model. The probe words list consists of 40 Italian words accurately chosen with regards to their frequency in child vocabulary repertoire and to their motor characteristics. The probe words list was developed according to the Motor Speech Hierarchy (MSH) [36], which is the developmental model of speech motor acquisition and control at the basis of the PROMPT motor speech framework. The model illustrates a hierarchical development of seven speech subsystems: Stage 1: Tone, Stage 2: Phonatory Control, Stage III: Mandibular Control, Stage IV: Labial-Facial Control, Stage V: Lingual Control, Stage VI: Sequenced movements and Stage VII: Prosody. The selected words vary in terms of motor complexity required at each stage of the hierarchy. The inclusion of a wide range of syllables and words shapes, ranging from easy, monosyllabic production with simple intersegmental transitions to multisyllabic words, allows data collection from children with limited motor speech skills as well as from children who are more verbal. A dedicated software, developed ad hoc by Khymeia s.r.l., will help the management of the speech tasks schedule (the probe words list) for the kinematic analyses, and will help to detect and delete repetition of non-optimal speech tasks contaminated by non-speech movements, non-pertinent conversations, etc.

### Additional outcome measures

*Diadochokinetic rate*: Diadochokinetic rate (DDK), assessed by a maximum performance task consisting in the fast repetition of a two- and/or three-syllable nonsense sequence (i.e. /pata/ or /pataka/) over 20 sec. (“count by time” method). The performance on this task will be compared to that of the same above described 40 TD children. The DDK rate is a robust speech measure that can provide information on the speech motor abilities of a speaker and on movement limitations (such as speech rate and the range of movements of the articulators), as well as elements for a differential diagnose among different speech sound disorders [37].

*Maximum phonation time*: the phonatory duration during the production of an open vowel (i.e. /a/) on a single expiration. This measure provides information on the functioning of speech breathing and of the laryngeal subsystem. Children with CP are reported to have shorter phonatory duration compared to TD children [38] due to an abnormally small vital pulmonary capacity [39–41]. Reduced expiratory volumes can affect the amount of speech movements that can be superimposed to phonation and, therefore, to speech expiration, thus determining a very effortful production of speech and, from the listener side, decreased intelligibility.

### Statistical analyses

Descriptive statistics and baseline differences between group will be investigated. Changes in clinical speech primary outcome measures (T0-T1) will be calculated to assess the short-term effects of PROMPT treatment versus standard care (waitlist group). Stability of the effects will be assessed at T2 in the PROMPT treated group. Secondary outcome kinematic speech measures will be also calculated at T0, T1 and T2 and included in the analyses. Covariates such as type of neurological impairment, type/severity of brain lesion and age, will be included in the analyses. Parametric or non-parametric statistics will be applied according to variables characteristics and distributions. Post-hoc adjustment will be applied for multiple comparisons when needed. Multivariate statistics will be finally performed. Data imputation will be considered according to characteristics and relationship of missing variables. Statistical significance will be considered at *p* < .05.

### Ethics

The present study was approved by the Paediatric Ethics Section of Tuscany Regional Ethics Committee on clinical trials (Italy), with study opinion registration number: 272/2020. Written consent will be obtained from parents of eligible infants, after being informed about the trial by the Principal Investigator or the research collaborators mentioned in the ethical committee approved protocol. Relevant protocol modifications will be promptly communicated to the abovementioned Ethical Committee for approval revision.

An appropriate electronic password-protected access system for the correct deidentification and anonymization of subjects and data collection and management will be used. Limited-access participants’ identifying information will be stored separately according to recent recommendations on sensitive data management and patients’ privacy.

## Discussion

This paper describes the study protocol of a waitlist-control RCT with the aim of evaluating the benefits of an intensive speech motor intervention such as PROMPT on speech and intelligibility in children with CP and the stability of the intervention benefits. A further objective is to assess the treatment induced modifications of the kinematic parameters of lips and jaw in speech tasks.

Despite communication being fundamental for participation and quality of life of children with CP, there is a lack of quality evidences about the efficacy of specific speech motor treatments in CP, compared to no intervention at all [11]. Model of adult acquired dysarthria, may not be effective as different mechanisms underlie speech motor disorders in congenital brain lesions. As a developing system, speech motor control requires the sensorimotor integrity, which may be disrupted by congenital or early acquired brain injury [15]. This negatively impacts the establishment and functioning of sensorimotor pathways and further affect activity-dependent synaptic plasticity. It can be hypothesized that early abnormalities determine dysfunctional evolution of motor control, including speech motor control [15, 16], which result in a multilevel impairment, comprising altered articulation, dysarthria and abnormal speech planning/programming [9]. Speech interventions based on the principles of motor learning are fundamental at early stages to optimize the mechanism of functional plasticity that are maximal during infancy and childhood [42].

PROMPT treatment is a well-established motor speech intervention for a variety of conditions [25]. It has several valuable characteristics that support its suitability in the CP field, being task specific for speech, child-initiated but still offering active tactile-kinesthetic assistance, and deliverable in a child friendly environment depending on the age and functional level, a fundamental aspect with regard to motivational enhancement and compliance to treatment. Nevertheless, there is a limited number of studies aimed at the implementation of feasible, cost effective, and targeted rehabilitation strategies, so appropriate interventions are often not provided at the most effective time and dose, eventually causing individuals with CP to face unnecessary challenges throughout lifetime.

## Data Availability

Datasets generated and/or analyzed during the current study are not yet publicly available as the recruitment is ongoing, but they can be available from the corresponding author on reasonable request. The Principal Investigators and the formal research collaborators will have access to the final trial dataset.

## Abbreviations

AAC: Augmentative Alternative Communication
ASHA: American Speech-Language-Hearing Association
CP: Cerebral palsy
DDK: Diadochokinetic rate
DST: Dynamic Systems Theory
ICS-ITA: Intelligibility in Context Scale - Italian version
IQ: Intelligence Quotient
MRI: Magnetic Resonance Imaging
MSH: Motor Speech Hierarchy
PROMPT: Prompts for Restructuring Oral Muscular Phonetic Targets
RCT: Randomized Controlled Trial
SLP: Speech and Language Pathologist
TD: Typically developing
VMPAC: Verbal Motor Production Assessment for Children
VSS: Viking Speech Scale
